# A Biological Signal-Based Stress Monitoring Framework for Children Using Wearable Devices

**DOI:** 10.3390/s17091936

**Published:** 2017-08-23

**Authors:** Yerim Choi, Yu-Mi Jeon, Lin Wang, Kwanho Kim

**Affiliations:** 1 Department of Industrial and Management Engineering, Kyonggi University, Suwon 16227, Korea; yrchoi@kgu.ac.kr; 2 Department of Industrial and Management Engineering, Incheon National University, Incheon 22012, Korea; jym9425@gmail.com; 3 Department of Library and Information science, Incheon National University, Incheon 22012, Korea

**Keywords:** child stress monitoring, wearable device, audio signal, heart rate, biological signal, machine learning

## Abstract

The safety of children has always been an important issue, and several studies have been conducted to determine the stress state of a child to ensure the safety. Audio signals and biological signals including heart rate are known to be effective for stress state detection. However, collecting those data requires specialized equipment, which is not appropriate for the constant monitoring of children, and advanced data analysis is required for accurate detection. In this regard, we propose a stress state detection framework which utilizes both audio signal and heart rate collected from wearable devices, and adopted machine learning methods for the detection. Experiments using real-world data were conducted to compare detection performances across various machine learning methods and noise levels of audio signal. Adopting the proposed framework in the real-world will contribute to the enhancement of child safety.

## 1. Introduction

Recently, a rapid growth in the number of instances of child abuse in nursery schools is being reported in Korea [1], and therefore, the necessity for real-time child monitoring is getting attention. Monitoring the stress state of children aged around three to five is particularly important, as their linguistic and physical abilities are immature, making it hard for their parents to be informed on their condition. In actual practice, a walkie-talkie for infants or CCTV in a nursery school are used for child monitoring, both of which have the limited coverage and provide only partial information related to the stress state of a child.

There is a limited number of stress detection studies directly conducted for infants or children. Most of them utilized audio signal collected from children for crying detection [2,3,4,5], which is relatively easy to obtain. Using only audio signal for monitoring a child is not practical, as distinguishing the voice or sound of a child in real circumstances, where many children are gathered in one place, is almost impossible. In such a condition, previously proposed stress detection methods would work poorly. For instance, the state of a child might be determined to be stressed using the previous methods even if the child is not crying when other children begin to cry. Therefore, utilizing biological signals—which has proven to be effective in previous studies on the stress detection of adults [6,7]—in addition to the audio signal will help reducing the false positives of the stress state detection of a child.

However, specialized equipment is required for the acquisition of most biological signals, and that equipment is sometimes too heavy or intrusive, making it unsuitable as a device for the constant monitoring of a child. Sensors for acquiring the brainwaves or electrodermal signal of the child need to be attached to the forehead or skin of a child, which is not possible in a real-world situation. On the other hand, heart rate can be collected using an unobtrusive device such as a wearable band with fairy accurate performances attributed to advents in the sensing technology.

To this end, we propose a stress detection framework for children using audio signal and biological signal (heart rate) acquired from a wearable device. Each child’s biological signal is collected by using a wearable device attached to their wrist. Then, the signal is transmitted to a server, and the child’s stress state is classified using a learning-based stress detection algorithm introduced in this paper. An alert is provided to the smart devices of their parents when the stress state of the child is detected.

Specifically, a three-step stress detection algorithm is introduced to provide accurate detection performances. First, raw audio signals are preprocessed to extract meaningful features, and are combined with heart rate. Second, features having high discriminative power for the stress and normal states are selected. Then, a classifier is learned from training data composed of the selected features, and stress states of children are determined using the classifiers.

The paper is organized as follows. The proposed stress monitoring framework and the stress detection algorithms are introduced in Section 2. In Section 3, the performances of the proposed framework are evaluated using real-world data, and the paper concludes in Section 4.

## 2. Literature Review

Table 1 shows the summary of previous research on stress state detection in terms of detection target, utilized method, and data. The previous studies are divided into two groups according to the target—child or adult.

Only a few studies have been conducted for infants or children. Audio signal collected from children has mainly been used in previous studies [2,3,4,5], attributed to the ease of data collection. Most studies adopted machine learning methods such as k-nearest neighbor [4] and hidden Markov model [3], since they can learn stress detection classifiers from data composed of multiple features without using explicitly defined rules or indices.

Unlike stress detection for children, diverse types of data such as biological signals have been used in the stress detection studies for adults. Biological signals include brainwave [10], electrodermal signal [8,9,10,11], and heart rate [6,7]. Accelerometer data [10] and respiration data [12] were also utilized. While most studies employed machine learning methods such as decision tree (DT) [7,8,10], naive Bayes (NB) [10], and support vector machine (SVM) [7,8,9,10,11,12] introduced indices for discriminating stress states.

The accuracy of stress detection performances in previous studies were around 80% to 90%. For instance, Healey [12] obtained an accuracy of around 97% by utilizing features from durations of 5 min. Setz and Sun [9,10] reported that their methods respectively yielded 82.8% and 91.0% accuracy by using SVM.

In addition, there are commercial devices for detecting stress states, such as iCalm [14] and ParentGuardian [15]. A summary comparison of the products and the proposed framework is shown in Table 2.

Both iCalm and ParentGuardian aim to detect the stress state of users like the proposed framework. However, iCalm is not only for infants or children but also for adults and utilizes diverse types of data by attaching the device to wrist and foot. ParentGuardian is generally for children, but is only tested for children with a special case. More importantly, both products do not utilize audio signal for detection.

## 3. Biological Signal-Based Stress Detection Framework for Children

### 3.1. Stress Detection Framework

**Figure 1:**
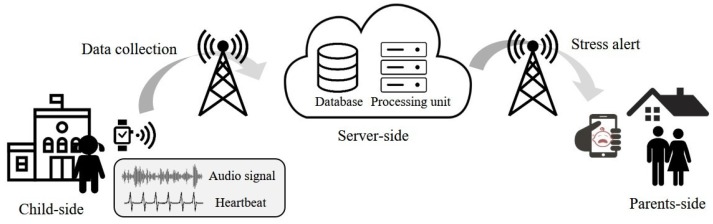
Framework of real-time stress monitoring for children using wearable devices.

In this section, we introduce a stress detection framework for children using wearable devices. Figure 1 shows the steps and elements of the proposed framework.

The proposed framework is composed of three elements: child-side, server-side, and parents-side. In the child-side, the audio signal and heart rate of a child are continuously sensed and saved in a wearable device attached to the child’s wrist. The collected data are transferred to the server at fixed intervals. On the server-side, the stress state of the child is determined by analyzing the transmitted data in a certain length. Then, on the parents-side, the detected state of the child is provided in real-time, and an alert is generated when the stress state is detected. Although the proposed framework is supposed to be real-time monitoring, there exists a latency since the collected data from a child is transmitted to a server at a fixed interval. However, the latency can be ignored by minimizing the interval, making the proposed framework similar to a real-time monitoring. Details of the stress detection algorithm are provided in Section 3.2.

### 3.2. Stress Detection Algorithm

#### 3.2.1. Overview

**Figure 2:**
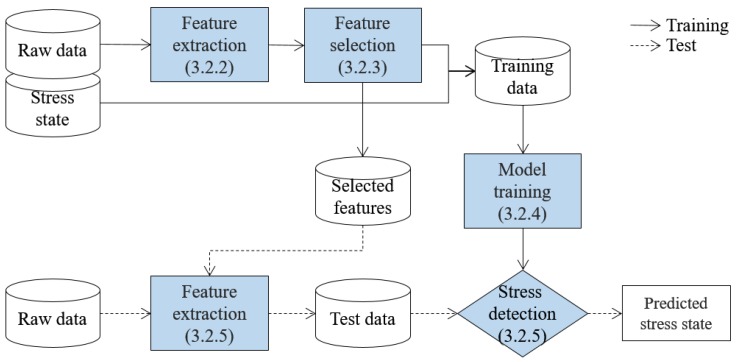
Overview of the learning-based stress detection algorithm.

After the audio signal and heart rate of a child are collected and transferred to the server, the stress state of the child is determined by using a learning-based stress detection algorithm. The overview of the algorithm is presented in Figure 2, which is composed of training and test phases. In Figure 2, solid and broken lines indicate the training and test phases, respectively, and shaded boxes indicate the detection steps, where the respective sections are noted in round brackets.

In the training phase, the stress detection method is developed after performing the following three steps. Firstly, meaningful features are extracted from the raw data. Audio signal is time-series data, and extracting meaningful features is one of the most important tasks for accurate classification [16]. Then, features with the highest discriminative power for stress state detection are selected, since irrelevant features can degrade the detection performance [17], and small number of features contributes to more efficient classification [18]. Lastly, a stress detection method is developed by training a machine learning method using the data composed of values of the selected features and a corresponding stress state label called training data.

In the test phase, the momentary stress state of a child is determined. Therefore, the test phase is repeatedly executed in real-time, unlike the training phase which is executed only for once. A child’s raw data collected in real-time is transformed to test data, which is composed of the values of the selected features in the training phase. Then, whether or not the child is in a stress state is determined by analyzing the test data by using the stress detection method from the training phase.

#### 3.2.2. Feature Extraction

It is important to extract meaningful features from the signal for accurate stress state detection, since we utilize audio signal which is time-series data. Diverse features are extracted from raw data using jAudio [19], which is an implementation of feature extraction algorithms for analyzing audio signals in java. Table 3 shows the 27 feature types provided in jAudio, which can be categorized into three groups according to the preprocessed data. Most feature types are calculated using the output of a discrete Fourier transform. Others are calculated using beat histogram or frequency information from the raw signal. Details of the feature types are provided in [19].

Since some feature types generate multidimensional vectors such as MFCC (mel-frequency cepstral coefficients) and beat histograms, while others generate single values such as RMS (root mean square) and spectral centroid, the total number of extracted features from jAudio is 136. The values of a feature for a certain duration are aggregated. Note that we utilized a general aggregator function including mean and standard deviation.

**Figure 3:**
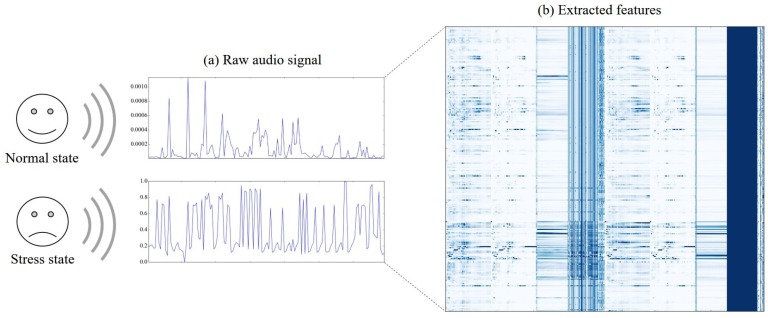
Example of the (**a**) raw audio signal and (**b**) extracted features in terms of the two stress states of a child: normal and stress.

Figure 3 shows (a) graphs of raw audio signal for the two states (normal (upper) and stress (lower)), and (b) a heatmap of the normalized values of the extracted features for every ten-second duration of the raw signal. Specifically, each column in the heatmap indicates one of the extracted features, and each row indicates one of the durations . For instance, a cell located in the third row and the fifth column is a value of the fifth feature calculated using the values in the third duration.

The amplitude of the raw signal in normal state is much smaller than that of the signal in stress state—about 1000 times smaller. It is noticeable that there are only a few features whose values have highly distinguishing patterns between stress and normal states, and most features show similar patterns. Therefore, selecting and utilizing the features with distinguishing patterns will generate good performance for the classification of the two stress states of children.

In addition to the extracted features of audio signal, we utilize the child’s heart rate for the detection. Particularly, we utilized the average of element heart rates for ten seconds as a heart rate, where an element heart rate is calculated using the duration of two consecutive heart beats, to obtain more accurate values for a short duration. Heart rate at the *i*-th duration is denoted by $h_{i}$, and calculated using Equation (1) for $0\leq j\leq n_{b}$.

(1) $$\begin{array}{c} h_{i}=\frac{1}{n_{b}+1}\sum_{j}\frac{60}{b_{j+1}-b_{j}}, \end{array}$$

where $n_{b}$ indicates the total number of heart beats at the *i*-th duration , and $b_{j}$ indicates the time when the *j*-th heart beat occurred.

#### 3.2.3. Feature Selection

For more accurate and efficient detection, feature selection was conducted to eliminate irrelevant features for the detection. Feature selection methods are categorized into filtering and wrapper [20] approaches. The filtering approach observes the relationship between values of a feature and their labels in terms of a certain criteria, and features are selected according to the scores of features calculated using the criteria. The wrapper approach repeatedly performs classification using different subsets of features in a predefined order, and compares their performance in order to select a subset with the best performance.

In the filtering approach, we adopted chi-square and information gain as the criteria, which were known to be the most effective for feature selection in comparison studies [21]. In the wrapper approach, we chose SVM as classifier, which is known to consistently show good performance [22] and be sensitive to whole features. We denote chi-square, information gain, and SVM wrapper as CHI, IG, and SVMW, respectively, in the following for simplicity.

1. Chi-square-based selection
  CHI utilizes the correlation between a feature and stress states by measuring the divergence of observed data from the expected distribution which assumes that the feature and labels are independent. The score for CHI is obtained as the sum of the square of the difference between observed value and expected value of a feature over the expected value. According to the score, the predefined number of features, denoted by $n_{f}$, are selected.
2. Information gain-based selection
  IG evaluates a feature by measuring the information gain with respect to the stress states. The score for IG is obtained as the difference in entropy when a feature is given or not. According to the score, the top $n_{f}$ features are selected.
3. SVM wrapper-based selection
  SVMW utilizes SVM as a classifier for the performance evaluation of subsets. For the subset generation, a best-first search is utilized, which known to work best for SVM [20]. Accuracy is adopted as an evaluation metric. According to the accuracy obtained by classification using subsets, the features included in the best subset are selected.

#### 3.2.4. Detection Model Training

Machine learning methods are trained for the stress state detection of children by using the selected features. Machine learning methods are widely utilized for classification and prediction problems such as energy consumption prediction [23], sentiment analysis [24], and scientific success prediction [25]. For the detection, we adopted the three most well-known machine learning methods: DT, NB, and SVM. Details of the models are provided in the following paragraphs.

We tried to detect the stress state, denoted by $y_{i}$, of a child for a duration, where *i* is an index of the duration and $y_{i}\in \left\{0,1\right\}$, 0 for normal state and 1 otherwise. Specifically, the length of a duration is predefined as 10 s. The value of the selected features for *i*-th duration is presented as a vector $X_{i}$ which is composed of $x_{i,j}$ where *j* is an index of the selected features and $1\leq j\leq n_{f}$. Therefore, a classifier is learned using training data composed of instances, denoted by $\left(X_{i},y_{i}\right)$ for $i=0,\cdots ,n_{d}$, where $n_{d}$ is the total number of durations.

1. Decision tree-based detection
  DT is a tree-shaped classifier where each node is composed of a feature and a corresponding classification value.When an instance is given to the root node, each node classifies the instance according to its feature and value pair. We utilized the C4.5 algorithm [26] which is an extension of ID3 [27] to handle continuous features as we examine time-series signals for the detection. The Gini index was adopted for the feature selection in each node.
2. Naive Bayes-based detection
  NB [28] uses Bayes’ rule for the computation of the probability of a given $X_{i}$ to be in $y_{i}$. A formal representation of the probability is shown in Equation (2). It assumes that, given a label, features are conditionally independent. The probabilities for features are estimated from data using maximum likelihood estimation.
  (2) $$\begin{array}{ccc} P(y_{i}|X_{i}) & = & \frac{P\left(X_{i}|y_{i}\right)P\left(y_{i}\right)}{P(X_{i})}\\ & \propto & P\left(x_{i,1},x_{i,2},\cdots ,x_{i,n_{f}}\right)P\left(y_{i}\right)\\ & = & \prod_{j}^{n_{f}}P\left(x_{i,j}|y_{i}\right)P\left(y_{i}\right). \end{array}$$
3. Support vector machine-based detection
  SVM [29] is one of the most well-known machine learning methods, and is widely applied to diverse domains (e.g., document classification) [30]. It finds the maximum margin among instances of normal and stress states. As a result, SVM shows relatively stable performances regardless of the number of training data and features. Equation (3) is a Lagrangian dual problem of the objective function of SVM. The optimal solution can be obtained by solving a quadratic programming.
  (3) $$\begin{array}{cc} \min & \frac{1}{2}\sum_{k}\sum_{l}y_{k}y_{l}\alpha_{k}\alpha_{l}\left(X_{k}\cdot X_{l}+\lambda \delta_{k,l}\right)-\sum_{l}\alpha_{l}\\ s.t. & 0\leq \alpha_{l}\leq C\\ & \sum_{l}\alpha_{l}y_{l}=0 \end{array}$$
  SVM has the advantage that it is able to classify data which are not linearly separated by using kernel function which maps a vector into a higher dimension. In this paper, we considered radial and linear kernels for comparison, and named them as SVM-R and SVM-L, respectively.

#### 3.2.5. Stress Detection

Using the trained methods, the stress state of a child at the $i^{\prime }$-th duration is determined. From the audio signal and heart rate collected at the $i^{\prime }$-th duration, $X_{i^{\prime }}$ is constructed according to the selected features. Then, the trained method determines the stress state of the child at the $i^{\prime }$-th druation, ${\hat{y}}_{i^{\prime }}$, which maximizes the probability using Equation (4).

(4) $$\begin{array}{c} {\hat{y}}_{i^{\prime }}=\arg\max_{y_{i^{\prime }}}P\left(y_{i^{\prime }}|X_{i^{\prime }}\right). \end{array}$$

## 4. Experiment

### 4.1. Stress Detection Device Prototype

**Figure 4:**
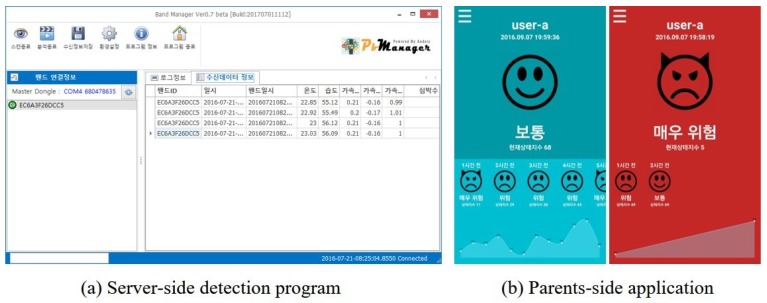
Prototype of (**a**) a stress detection program in server-side and (**b**) a monitoring application in parents-side.

**Figure 5:**
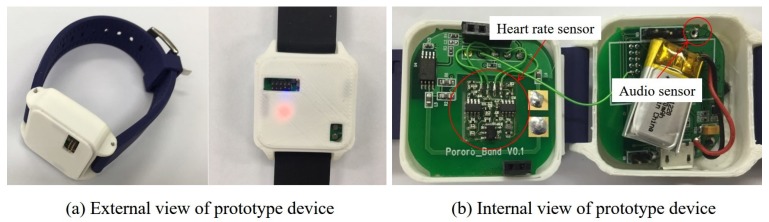
(**a**) External view and (**b**) internal view of the prototype of the wearable device, where the audio signal and heart rate of a child are collected.

A prototype framework was implemented for the evaluation of the proposed framework. Figure 4 and Figure 5 show the child-side, server-side, and parents-side elements in the framework. The external and internal views of the prototype device are presented in Figure 5a,b, respectively. Note that the device is a prototype and that the wearing sensation was not considered. Inside the device, there are two sensors: a microphone for acquiring audio signal and a heart rate sensor which collects heart rate by attaching to the inner side of the wrist.

Figure 4a is a snapshot of the stress detection program which collects test data from a wearable device with a time-stamp and detects the stress state of a child at that time using the trained stress detection method. Then, the results are sent to the application on the parents-side, reporting the stress state of their child. Figure 4b shows a screenshot of the application when a child is in normal state (left) or in stress state (right).

### 4.2. Experiment Settings

We have conducted experiments to observe the performances and characteristics of the stress detection method in the proposed framework. For the evaluation, we have collected real-world audio signal and heart rate. The collected data were divided into instances with length of ten seconds. The total number of instances was 262, where the numbers of the instances in stress and normal states were, respectively, 138 and 124.

Note that the dataset utilized in the experiments was syntactically generated, since collecting data from children in a stress state may cause an ethical issue. The dataset was generated by combining audio signal obtained from [31] which is a collection of sounds from a number of children and heart rate obtained from six subjects to conform with the stress and normal states. Moreover, we assumed that children who are crying are in a stress state and marked the data as a stress state. For example, audio signal and heart rate of a child who was having fun while running and screaming were used as one of the instances in normal state. For evaluation, we have employed *k*-fold cross validation, where *k* was set 10 to minimize randomness.

To examine the effect of diverse settings on the detection performance, we have evaluated the performances of the method according to the utilization of heart rate, the number of the selected features, and the noise level. Specifically, the audio signal with noise was considered to investigate the robustness of the proposed method in real circumstances, where other sounds exist. We utilized Adobe Audition CC [32] to generate the audio signal with noise, where white noise is generated with the intensity of the noise on a scale of 2 to 40. As the intensity gets higher, the noise becomes more erratic, harsher, and louder. Therefore, the noise level indicates the strength of white noise added to the original audio signal, and we considered six levels: 0, 5, 10, 15, 25, and 40.

We adopted accuracy as an evaluation measure. Accuracy is one of the most widely utilized metrics for classification problems [30], and is defined as the ratio of the number of instances which are correctly classified over the number of all instances, as shown in Equation (5).

(5) $$\begin{array}{c} Accuracy=\frac{TP+TN}{TP+FP+FN+TN}, \end{array}$$

where TP (true positive), FP (false positive), FN (false negative), and TN (true negative) respectively indicate the numbers of instances when the predicted and the actual states are stress, the predicted state is stress while the actual state is normal, the predicted state is normal while the actual state is stress, and the predicted and actual states are normal (Table 4).

In addition to the accuracy, we adopted recall and precision for detailed evaluation. Recall indicates the sensibility of a model and is calculated as the ratio of the number of instances which are correctly classified over the number of instances which are actually in stress state, as per Equation (6).

(6) $$\begin{array}{c} Recall=\frac{TP}{TP+FP}. \end{array}$$

Precision is calculated as the ratio of the number of instances which are correctly classified over the number of instances which are predicted as stress state, as per Equation (7).

(7) $$\begin{array}{c} Precision=\frac{TP}{TP+FN}. \end{array}$$

### 4.3. Experimental Results

#### 4.3.1. Feature Selection

Features with high discriminative power were selected among the features extracted from the audio signal. We considered three methods—CHI, IG, and SVMW—for the feature selection. Table 5 shows the top five selected features according to the three methods.

It is noticeable that there were features which commonly appeared across the selection methods, such as MFCC overall standard deviation and heart rate. MFCC-related features were most frequently selected for all selection methods, and among them, MFCC overall standard deviation showed highest ranks. Heart rate ranked the first for both CHI and IG, and although heart rate was not included in the top five features of SVMW, it also ranked the ninth for SVMW. Moreover, the rank of heart rate for SVMW got higher as the noise level increased. Heart rate ranked ninth, sixth, fifth, and first by SVMW according to the noise level of 0, 5, 10, and 40, respectively.

While the top five selected features of CHI and IG were similar, those of SVMW differed from those of CHI and IG. This is explained by the difference in the concept of the filtering and wrapper approaches. CHI and IG basically examine the relationship between features and labels (particularly correlation), while SVMW heuristically tests the subsets of features for the detection. Therefore, SVMW incorporates the interaction among features, resulting in more diverse features than CHI and IG.

**Figure 6:**
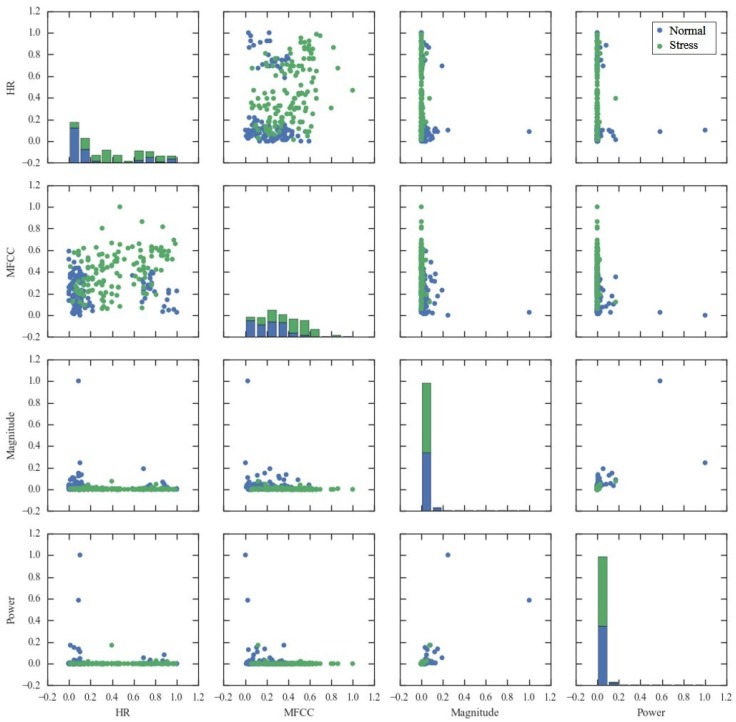
Scatter matrix of four selected features which appear most frequently among the feature selection results by the three methods (CHI, IG, and SVMW). HR: heart rate; Magnitude: magnitude spectrum overall average; MFCC: MFCC overall standard deviation; Power: power spectrum overall average.

Moreover, to examine the discriminative power of the selected features, a scatter matrix is provided in Figure 6. We considered the four features which appeared most commonly across the selection methods, including heart rate (HR), MFCC overall standard deviation (MFCC), magnitude spectrum overall average (Magnitude), and power spectrum overall average (Power). In Figure 6, diagonal plots show the histogram of stress states according to the value of each feature, and non-diagonal plots show the scatter plot of feature pairs.

Scatter plot of HR and MFCC seems most discriminative for stress and normal states. When a child is in normal state, smaller values of MFCC and extreme values of HR are expected. In terms of Power and Magnitude, most instances have small values, while some of normal state have extremely large values.

#### 4.3.2. Performance Comparison

**Figure 7:**
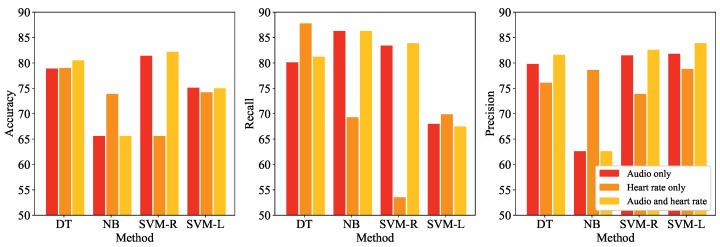
Performance comparison results of the proposed framework in terms of the utilized data, audio only, heart rate only, and audio and heart rate together, and the adopted methods—decision tree (DT), naive Bayes (NB), SVM with radial kernel (SVM-R), and SVM with linear kernel (SVM-L)—according to the evaluation measures (**left**) accuracy; (**middle**) recall; and (**right**) precision.

We conducted three performance comparison experiments. First, state detection performances according to the employed data and method were evaluated. Figure 7 shows the accuracy of the four detection methods—DT, NB, SVM-R, and SVM-L— according to the utilized data, audio signal only, heart rate only, and both audio signal and heart rate in terms of accuracy, recall, and precision. Note that all extracted features were employed in this experiment.

The best accuracy was 82.18 when both data was utilized and SVM-R was employed, while the worst one was 65.27 when only heart rate was utilized and SVM-R was employed. In terms of the utilized data, for the three methods except for NB, the best accuracy was obtained when both audio signal and heart rate were employed. DT and SVM-R performed the best in terms of the employed methods, as the average accuracies of methods DT, NB, SVM-R, and SVM-L across the utilized data were 79.47, 68.37, 76.40, and 74.77, respectively. The low accuracy of NB implies that there may exist correlations among features which NB ignores. The differences between recall and precision of NB and SVM-L are large, while those of DT and SVM-R are relatively small, implying that NB tends to classify instances as stress states excessively whereas SVM-L does the opposite. Utilizing heart rate contributed to conservative classification, since precisions were higher than recalls for most cases where heart rate was utilized. Table 6 shows the results of *t*-test conducted on the accuracies obtained by performing 10-fold cross-validation. Except for the comparison between DT and SVM-R when only audio signal was utilized, alternative hypothesis is accepted at significance level of 0.05.

**Figure 8:**
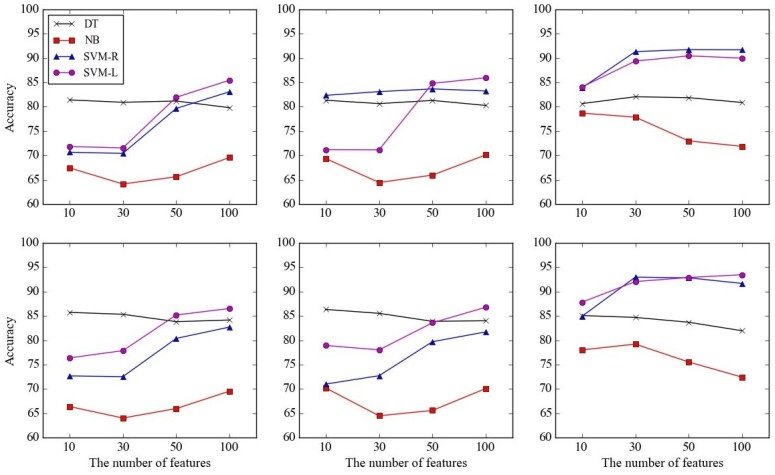
Performances of the proposed framework using the selected features according to the number of selected features and detection methods in terms of data utilized: (**upper**) audio only and (**lower**) audio and heart rate, and feature selection methods: (**left**) CHI , (**middle**) IG , and (**right**) SVMW.

Second, the detection performances of the proposed framework using the selected features are shown in Figure 8. Performances of the four methods—DT, NB, SVM-R, and SVM-L—are presented according to $n_{f}$, 10, 30, 50, and 100, and the feature selection methods, CHI (left), IG (middle), and SVMW (right). The upper plots in Figure 8 show the accuracies when only audio signal was used, and the lower plots show those when both audio and heart rate were used. For comparison purposes, detection accuracies using all features are provided on the right-side of the graphs.

The best accuracy was 93.47 when the feature selection method, detection method, and $n_{f}$ were SVMW, SVM-L, and 100, respectively, while the worst accuracy was 64.01 when the feature selection method, detection method, and $n_{f}$ were CHI, NB, and 30, respectively. Overall, detection performances were better when feature selection was conducted, since irrelevant features were removed from the training dataset. This conforms with the well-known fact that the performances of a machine learning method degrade when irrelevant features are utilized. The average accuracies of the feature selection methods CHI, IG, and SVMW across the other factors were 76.37, 77.54, and 84.65, respectively, implying that the wrapper approach outperforms the filtering approach.

Moreover, it is noticeable that as $n_{f}$ increases the accuracies of SVM-R and SVM-L increase, while they remain still or sometimes decrease for DT and NB. This can be explained by the fact that NB is robust to the irrelevant features, and DT internally selects good features during training.

**Figure 9:**
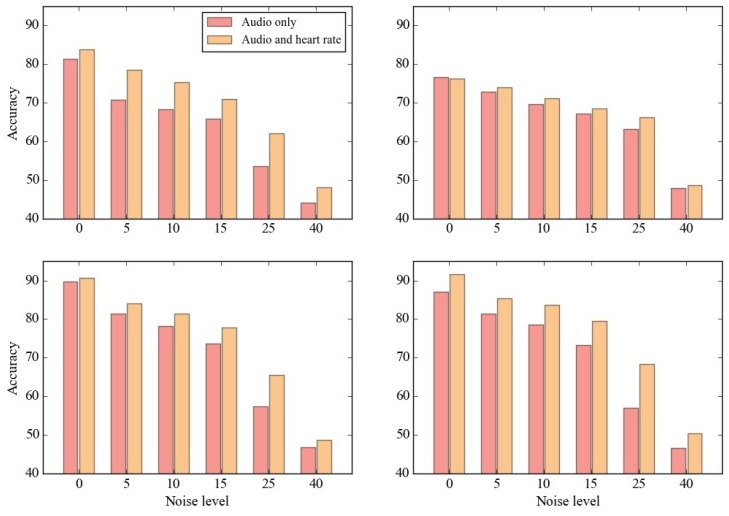
Performances of stress detection using the four detection methods: (**upper left**) DT , (**upper right**) NB , (**lower left**) SVM-R , and (**lower right**) SVM-L, according to the noise level (5, 10, 15, 25, 40) and utilized data (audio only and both audio and heart rate).

Last, to evaluate the performance of the proposed method in a real-world situation, we conducted detection on data with noise. Figure 9 shows the accuracies of the four methods—DT (upper left), NB (upper right), SVM-R (lower left), and SVM-L (lower right)— according to the five noise levels, 5, 10, 15, 25, 40, and utilized data with audio only and with both audio and heart rate. Note that SVMW—which performed the best in the previous experiment—was adopted as a feature selection method, and the accuracies were averaged a across $n_{f}$, 10, 30, 50, and 100.

Overall, it was observed that the accuracies decreased as the noise level increased, as expected. The accuracy decrements were much larger between 5 and 15 than between 15 and 25. Particularly, when heart rate was utilized together with audio signal, the performance was more robust than using only audio signal, implying that utilizing heart rate not only improves accuracy but also makes the method robust to environment.

## 5. Conclusions

In this paper, we proposed a stress detection framework for children using audio signal and heart rate acquired from a wearable device. The proposed framework is composed of three parts: child-side, where data is collected; server-side, where the stress detection is conducted; and parents-side, where detection results are presented. The stress detection algorithm is divided into two phases: training phase, where detection method is developed; and test phase, where the real-time stress detection is conducted.

Both audio signal and heart rate of a child are utilized for the stress detection. Three feature selection methods—CHI, IG, and SVMW—were employed to determine the most effective features from raw audio signal, and four detection methods—DT, NB, SVM-R, and SVM-L—were adopted for performance comparison. SVMW-based feature selection and SVM-L showed the best performance. Moreover, the accuracy of the proposed framework using audio signal with diverse levels of noise was evaluated to examine the performances of the proposed method in a real situation. In conclusion, eliminating irrelevant features improved the performances, and utilizing both heart rate and audio signal enhanced the performance and made the method more robust to noise.

The advantages of the proposed framework over the previous studies are as follows. First, the proposed method is more robust to noise in the audio signal by utilizing heart rate in addition to audio signal, so it is able to identify the state of a target child even if the child is in noisy circumstances. Second, the proposed method may detect the stress state of a child with special conditions such as autism by analyzing the heart rate of the child along with the audio signal, even if a child does not make any noise. However, a specially trained model which uses data collected from the children with a special condition to reflect the characteristics of those children would work the best. Third, no additional equipment except for a wrist band is required for the detection. Complex models using diverse features and highly computational methods may perform better in an experimental environment, but they are not practical.

For future work, we plan to extend our research in terms of utilized data, features, and methods. We will conduct a large-scale experiment, and utilize additional signals such as accelerometer and electrodermal data, which were effective in previous work for adults. Lastly, privacy issues should be considered for the adoption of the proposed method since it utilizes the human-generated data. Therefore, we will adopt an on-device method, where data analysis is conducted only in a device without sending private data to the outside, in order to resolve the privacy problem.

## Figures and Tables

**Table 1:** Summary of previous research on stress detection in terms of their target, utilized method, and data.

Target	Method	Data	References
Child	Machine learning	Audio signal	[2]
			[3]
			[4]
			[5]
Adult	Machine learning	Electrodermal data	[8]
			[9]
			[10]
		Heart rate	[6]
			[7]
		Audio signal etc.	[8]
			[10]
	Index-based	Electrodermal data etc.	[11]
			[12]
			[13]

**Table 2:** Summary of the commercial stress detection devices in terms of their target, utilized data, and wearable design.

	Proposed Framework	iCalm	ParentGuardian
Target	Infants, children	Infants, children, adults	ADHD children
Data	Audio signal, Heart rate	Temperature Motion Electrodermal data Blood volume pulse	Electrodermal data
Wearable design	Wrist	Wrist, foot	Wrist

**Table 3:** List of feature types provided in jAudio [19]. FFT: fast Fourier transform; MFCC: mel-frequency cepstral coefficient; RMS: root mean square.

Power Spectrum	Spectral Flux	Fraction of Low-Energy Frames
Magnitude Spectrum	Partial-Based Spectral Flux	Linear Prediction Filter Coefficients
Magnitude Spectrum Peaks	Method of Moments	Beat Histogram
Spectral Variability	Area Method of Moments	Strongest Beat
Spectral Centroid	MFCC	Beat Sum
Partial-Based Spectral Centroid	Area Method of Moments of MFCCs	Strength of Strongest Beat
Partial-Based Spectral Smoothness	Zero Crossings	Strongest Frequency via Zero Crossings
Compactness	RMS	Strongest Frequency via Spectral Centroid
Spectral Roll-off Point	Relative Difference Function	Strongest Frequency via FFT Maximum

**Table 4:** Confusion matrix of instances in terms of predicted and actual states: stress and normal.

		Predicted State	
		Stress	Normal
Actual state	Stress	True positive (*TP*)	False positive (*FP*)
	Normal	False negative (*FN*)	True negative (*TN*)

**Table 5:** Top five selected features according to the three feature selection methods (chi-square, CHI; information gain, IG; and support vector machine wrapper, SVMW), according to their ranks.

Rank	CHI	IG	SVMW
1	Heart rate	Heart rate	MFCC overall standard deviation
2	MFCC overall standard deviation	MFCC overall standard deviation	Spectral flux overall standard deviation
3	Magnitude spectrum overall average	Magnitude spectrum overall average	Strongest beat overall average
4	Power spectrum overall average	MFCC overall average	Magnitude spectrum overall standard deviation
5	MFCC overall average	Power spectrum overall average	Compactness overall average

**Table 6:** Results of *t*-test for accuracies obtained by performing 10-fold cross validation according to the utilized models and data.

Data	Model	*t*	*p*-Value	Mean Difference
Audio only	NB and DT	−30.87	0.00	−14.89
	NB and SVM-L	−24.06	0.00	−9.46
	NB and SVM-R	−33.86	0.00	−16.60
	DT and SVM-L	8.23	0.00	5.42
	DT and SVM-R	−2.10	0.07	−1.72
	SVM-L and SVM-R	−13.00	0.00	−7.14
Heart rate only	NB and DT	−25.88	0.00	−5.15
	NB and SVM-L	−3.25	0.01	−0.35
	NB and SVM-R	87.20	0.00	8.32
	DT and SVM-L	26.42	0.00	4.81
	DT and SVM-R	71.24	0.00	13.47
	SVM-L and SVM-R	76.02	0.00	8.67
Audio and heart rate	NB and DT	−26.65	0.00	−13.28
	NB and SVM-L	−19.11	0.00	−9.54
	NB and SVM-R	−31.43	0.00	−15.80
	DT and SVM-L	4.76	0.00	3.74
	DT and SVM-R	−3.10	0.01	−2.52
	SVM-L and SVM-R	−7.84	0.00	−6.26
